# TPA-ConvNeXt: Trigonometric Phase Attention for Robust Retinal Disease Classification Across Fundus and OCT

**DOI:** 10.3390/bioengineering13070781

**Published:** 2026-07-07

**Authors:** Nebras Sobahi, Orhan Atila, Taha Özçelik, Abdulkadir Sengur

**Affiliations:** 1 Department of Electrical and Computer Engineering, Faculty of Engineering, King Abdulaziz University, Jeddah 21589, Saudi Arabia; nsobahi@kau.edu.sa; 2 Department of Electrical-Electronics Engineering, Faculty of Technology, Firat University, 23100 Elazig, Türkiye; ksengur@firat.edu.tr; 3 Department of Electrical-Electronics Engineering, Faculty of Engineering, Bingol University, 12000 Bingol, Türkiye; sozcelik@bingol.edu.tr

**Keywords:** Trigonometric Phase Attention, ConvNeXt, layer-wise learning rate decay, fundus images, OCT images, HYAMD, OCT2017

## Abstract

This study proposes TPA-ConvNeXt, a ConvNeXt-Small-based deep learning architecture for retinal image classification using a phase-based trigonometric attention mechanism. The proposed Trigonometric Phase Attention (TPA) block recalibrates feature maps through learnable phase and amplitude modulation derived from channel-wise and spatial context. In addition, a stable fine-tuning strategy combining layer-wise learning rate decay (LLRD), linear warmup, and cosine annealing is employed to adapt pretrained backbones to medical image data. The method was evaluated on both fundus and optical coherence tomography (OCT) datasets. On the HYAMD fundus dataset, 5-fold cross-validation yielded a mean Macro F1 of 0.8940 ± 0.0403 and an ROC-AUC of 0.9449. External fundus validation further demonstrated cross-dataset robustness, achieving 0.9312 Macro F1 and 0.9803 ROC-AUC when trained on HYAMD and tested on AMDNet23. In OCT experiments, the model achieved Macro F1 scores of 0.9790 on MAK1_OCT, 0.9387 on OCTDL, and 0.9989 on the cleaned OCT2017 benchmark test set after MD5 duplicate removal. Ablation results showed that warmup contributed most strongly to stable optimization, while the proposed TPA block provided a smaller but consistent performance gain. Additional statistical analysis across the five HYAMD folds showed that TPA-ConvNeXt achieved comparable performance to the ConvNeXt-Small baseline, with a small numerical accuracy difference that did not reach statistical significance. Grad-CAM visualizations indicated that the model focused on clinically relevant retinal regions, and complexity analysis showed that the full model required 51.82 M parameters and 17.41 GFLOPs, adding only 2.36 M parameters and 0.02 GFLOPs over the ConvNeXt-Small baseline. These findings suggest that TPA-ConvNeXt provides a robust and generalizable framework for retinal image classification across both fundus and OCT modalities.

## 1. Introduction

Age-related macular degeneration (AMD) has emerged as a major cause of permanent blindness, primarily among the elderly population [1,2]. Hence, it becomes critical to make an early diagnosis and accurately determine the stages of AMD to control the progression of the disease and make appropriate treatment decisions. In the clinical setting, color fundus photography and optical coherence tomography have been identified as the two most commonly employed imaging techniques for the evaluation of AMD [3]. Images obtained by fundus photography provide a two-dimensional view of the retinal surface, whereas OCT provides a detailed three-dimensional view of the retinal surface. Therefore, it can be inferred that automated classification systems would provide consistent results for both modalities.

Recent advances in artificial intelligence, particularly in deep learning, have significantly improved the ability to analyze retinal images [4]. Convolutional neural networks (CNN) have been found to achieve good results in the classification of fundus images as well as OCT images. Recent studies have explored vision transformers and hybrid models in the classification of AMD [5]. Traditional CNN architectures such as ResNet, DenseNet, and EfficientNet have been found to be successfully adapted from natural images to medical images, achieving good results in the classification of retinal images [6,7,8]. However, such models are often pre-trained on natural images, which may not always capture the intricate structural differences seen in medical images. Moreover, small sample sizes, imbalance, and variability in image distributions are some of the limitations faced.

Transformer-based and hybrid approaches have received significant attention in retinal image analysis. Vision Transformer (ViT) [9] and Swin Transformer [10] are capable of learning long-range dependencies in images, which may be beneficial for large-scale image classification tasks. However, the increased number of parameters and tendency to overfit may limit the usability of transformer-based approaches. In such a scenario, ConvNeXt [11] has been found to be a robust solution by incorporating multiple transformer-based design elements within a convolutional network.

While many existing studies have reported competitive results for AMD detection, most have focused on a single imaging modality, a single dataset, or limited internal validation settings [12]. These issues are especially important in medical image analysis, where class imbalance, dataset shift, and acquisition variability can strongly affect model performance [13]. Such issues are especially critical in medical image analysis, where dataset variability can have a significant impact on model performance [14]. To address these limitations, we propose TPA-ConvNeXt, a ConvNeXt-Small-based architecture equipped with a phase-based trigonometric attention mechanism. The proposed TPA block performs nonlinear feature modulation using learnable phase and amplitude terms derived from channel-wise and spatial context. Importantly, the term “phase” is used here in a classical signal-modulation sense; the method does not involve quantum computation, qubits, quantum measurement, or quantum hardware [15].

In addition to the architectural contribution, we employ a fine-tuning strategy designed for stable adaptation of pretrained backbones to retinal imaging data. This strategy combines layer-wise learning rate decay (LLRD), linear warmup, and cosine learning rate annealing. Our ablation analysis shows that optimization-level stabilization and architecture-level feature modulation make complementary contributions, with warmup providing the strongest gain and TPA contributing a smaller but consistent improvement.

The proposed framework is evaluated across both fundus and OCT modalities. Specifically, we report 5-fold cross-validation results on HYAMD, external fundus validation using AMDNet23, and additional experiments on multiple OCT datasets collected under different clinical and benchmark settings. The main contributions of this study are as follows:

(1) We propose a phase-based trigonometric attention block that modulates ConvNeXt features through learnable phase and amplitude interactions.
(2) We analyze a stable fine-tuning strategy based on layer-wise learning rate decay, warmup, and cosine scheduling for medical image transfer learning.
(3) We evaluate the proposed model across both fundus and OCT modalities, including external fundus validation and cross-dataset experiments.
(4) We provide ablation, statistical comparison, explainability analysis, and computational cost assessment to better characterize the behavior of the proposed framework.

## 2. Related Works

### 2.1. Deep Learning for AMD Detection from Fundus Images

Deep learning has become a central tool in automated AMD detection from fundus photographs. García-Floriano and Ventura-Molina proposed a transfer learning framework for AMD detection using fundus images collected from the ODIR5k database and the SAMRH projects. Their study compared several pre-trained CNN models, including AlexNet, VGG16, VGG19, GoogLeNet, InceptionV3, SqueezeNet, and Xception, and reported the best validation accuracy of 0.91 with Xception [12]. Although the study demonstrated the potential of transfer learning for AMD detection, its relatively small sample size, single-center design, and lack of external validation limited its clinical generalizability.

Chen et al. developed an AI-based framework for AMD screening using a large multi-center fundus image dataset collected from multiple devices and institutions [16]. Their convolutional neural network, built on a fine-tuned pre-trained backbone, achieved an AUC of 0.93, an accuracy of 0.84, and an F1-score of 0.86. The use of an external cohort was an important step toward improving generalization; however, the overall classification performance remained moderate, suggesting that robust AMD screening from heterogeneous fundus images remains challenging.

Explainability has also become an important topic in fundus-based AMD detection. Osa-Sanchez et al. proposed an explainable artificial intelligence framework based on a cascaded classification strategy and evaluated CNN, MLP, and transformer-based classifiers on the University of Pennsylvania’s CATT dataset [17]. Their best cascaded CNN model achieved an accuracy of 94.19% and an F1-score of 93.98%, while SHAP and LIME were employed to improve interpretability. However, the dataset size remained limited and the study relied on single-center data, which may restrict broader applicability.

Several studies have also explored hybrid or enhanced preprocessing pipelines for fundus-based AMD detection. Ali et al. introduced AMDNet23, a hybrid CNN-LSTM model trained on a four-class dataset compiled from multiple public fundus datasets [18]. By combining enhanced preprocessing, including CLAHE and gamma correction, with data augmentation, their approach achieved an accuracy of 96.5%. Similarly, Bao et al. proposed AttResAMD, an attention-based deep learning framework built on ResNet18 with a CBAM module to classify normal, dry AMD, and wet AMD from fundus photographs [14]. Their model achieved 93.3% accuracy on internal test data and 86.5% accuracy on an external validation dataset, while also outperforming ophthalmologists in some diagnostic comparisons. Nevertheless, the absence of k-fold cross-validation and the limited treatment of class imbalance remain important limitations.

Fundus imaging has also been used beyond AMD-specific classification. Hu et al. introduced FundusNet, an ensemble-based framework for detecting multiple neurodegenerative and ocular diseases from fundus images using the UK Biobank dataset [19]. Although their reported AUC for AMD was 0.75, the work highlighted the broader potential of fundus-based screening. More recently, lightweight and deployable models have also been proposed. Castro et al. presented a Swin Transformer-based system for grading AMD severity from fundus images, achieving an overall accuracy of 84.76% across five severity categories [20]. While such approaches are promising for web-based deployment, performance remains limited for fine-grained AMD staging.

### 2.2. OCT-Based AMD Classification

OCT imaging provides detailed cross-sectional information about retinal layers and is widely regarded as one of the most informative modalities for AMD diagnosis. A large body of research has therefore focused on OCT-based AMD classification using deep learning models. In an earlier benchmark study, Kermany et al. demonstrated the effectiveness of transfer learning with InceptionV3 on a large OCT dataset, reporting an accuracy of 96.53% [21]. Their work established OCT as a highly promising modality for automated retinal disease classification. However, the use of large pre-trained models may introduce computational overhead and may not always be ideal for practical deployment.

Subsequent studies explored more specialized OCT architectures. He et al. proposed a multipath CNN framework for AMD detection using OCT images and reported high classification accuracies across several datasets [22]. Celebi et al. used Capsule Networks on private and public OCT datasets and obtained strong performance, especially for early AMD detection [23]. Hu et al. introduced a hierarchical classification model for dry AMD using multiple CNN backbones such as EfficientNetV2, DenseNet169, Xception, and Normalizer-Free ResNet50, achieving a best F1-score of 92.08% [24]. Although these studies reported promising results, they often focused on OCT alone and were generally evaluated in modality-specific settings.

Other studies investigated more complex architectures or ensemble strategies. Chen et al. used an ensemble of AlexNet, VGG16, InceptionV3, ResNet50, and DenseNet for OCT-based lesion screening and reported an accuracy of 98.5% and an F1-score of 97.7% [25]. Das et al. proposed a multi-scale deep feature fusion framework and achieved 99.6% accuracy on the UCSD OCT dataset [26]. While such methods can provide excellent classification performance, they often require higher computational cost and may rely heavily on architecture complexity or manually tuned loss functions.

The recently published Diagnostics 2024 study by Yusufoğlu et al. introduced a CNN-based OCT model combining modified Inception modules, depthwise squeeze-and-excitation blocks, and ConvMixer components for AMD classification [27]. On their private OCT dataset of 2316 images, the proposed model achieved 97.98% accuracy and a 97.86% F1-score, while also achieving perfect performance on the public Noor dataset. This study demonstrated that carefully designed CNN architectures remain highly competitive for OCT-based AMD diagnosis.

### 2.3. Multimodal and Hybrid Approaches

More recently, researchers have begun to investigate multimodal learning strategies that combine fundus and OCT images for AMD diagnosis. Annamalai et al. proposed AMD-MMViT, a multimodal vision transformer framework using both OCT and fundus images [13]. Their approach combined modality-specific feature extraction with attention-based multimodal fusion and reported 98.1% accuracy using only fundus images, 99.2% using only OCT images, and 98.65% using both modalities. While these results are promising, the use of OCT and fundus images from different patients and the reliance on a simple 80–20 random split raise concerns regarding the robustness of the reported performance.

Multimodal approaches are attractive because fundus and OCT provide complementary retinal information. However, the practical challenges of assembling well-aligned multimodal datasets, along with validation issues, remain substantial. Consequently, robust single-backbone architectures that can generalize across different datasets and imaging modalities remain highly relevant.

### 2.4. Attention Mechanisms and Fine-Tuning Strategies

Attention mechanisms have become a major component of modern retinal image classification systems. Channel attention methods such as squeeze-and-excitation (SE) [15] blocks and combined channel–spatial modules such as CBAM [28] have been widely applied in medical imaging [29]. However, most of these approaches rely on relatively simple linear or sigmoid-based recalibration strategies. Recent work suggests that more expressive feature modulation methods, including frequency- or phase-aware mechanisms, may be beneficial for capturing subtle retinal structures. Nevertheless, phase-based attention remains largely underexplored in AMD classification.

Another important but often overlooked factor is the fine-tuning strategy used for adapting pre-trained backbones to medical datasets [30]. Layer-wise learning rate decay (LLRD) allows earlier layers to be updated more conservatively than later layers, thereby reducing the risk of disrupting useful pre-trained representations [31]. Similarly, warmup schedules can improve optimization stability by preventing abrupt parameter updates during the early epochs of training [32]. Despite their known benefits, these strategies are rarely analyzed in a systematic manner in AMD classification studies. Most works report final performance without quantifying the individual contributions of such optimization components.

### 2.5. Positioning of the Present Study

Compared with the existing literature, the present study differs in several key aspects. First, rather than relying solely on conventional channel or spatial attention, we propose a phase-based trigonometric attention mechanism that performs nonlinear trigonometric modulation of feature maps. Second, we combine this architectural contribution with a carefully designed fine-tuning strategy based on LLRD and warmup, and we explicitly analyze the contribution of these components through ablation experiments. Third, the proposed model is evaluated not only on a fundus dataset but also on multiple OCT datasets, allowing us to examine cross-dataset and cross-modality generalization in a more comprehensive manner than most previous studies.

Taken together, these characteristics position TPA-ConvNeXt as a contribution that addresses both architectural innovation and training stability, while also emphasizing robustness across heterogeneous retinal imaging settings.

## 3. Proposed Method: TPA-ConvNeXt

This section presents the proposed TPA-ConvNeXt architecture in detail. First, the overall network design is introduced. Then, the mathematical formulation of the Trigonometric Phase Attention (TPA) block is described. Finally, the training strategy is explained.

### 3.1. Overall Architecture

TPA-ConvNeXt is a phase-based trigonometric attention architecture built on top of the ConvNeXt-Small backbone. The model preserves the hierarchical four-stage feature extraction design of ConvNeXt and inserts the proposed Trigonometric Phase Attention (TPA) block after each stage. In this way, feature maps at different scales are recalibrated through a phase-based modulation mechanism.

**Figure 1:**
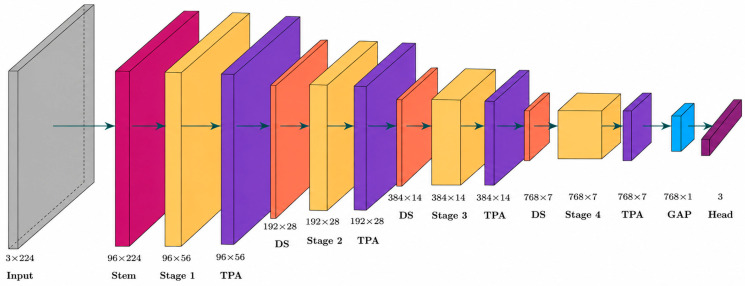
Overall architecture of TPA-ConvNeXt. The figure shows the ConvNeXt backbone, the Trigonometric Phase Attention (TPA) blocks inserted after each stage, and the final classification head.

Given an input image $X\in R^{B\times 3\times 224\times 224}$, the image is first processed by the stem layer to obtain the initial feature representation. It is then passed sequentially through Stages 1 to 4. At the output of each stage, the resulting feature map

(1) $$X_{l}\in R^{B\times C_{l}\times H_{l}\times W_{l}}$$

is fed into a TPA block, where phase-based modulation is applied. After the final stage, global average pooling is used to produce a compact feature vector, which is then forwarded to a fully connected classification layer. The overall architecture of the proposed model is illustrated in Figure 1.

### 3.2. Trigonometric Phase Attention (TPA) Block

The proposed TPA block modulates feature maps through phase components derived from both channel-wise and spatial contexts. In this work, the term “phase” refers to a learnable angular variable used in a classical trigonometric modulation function. The proposed TPA block is fully implemented within a standard deep neural network and does not involve quantum computation, qubits, quantum measurement, or quantum hardware. Let the input feature map be defined as:

(2) $$X\in R^{B\times C\times H\times W}$$

The channel phase component is obtained by combining two context vectors generated through global average pooling and global max pooling. This combined representation is passed through two separate fully connected layers to produce the channel phase $\theta_{c}$ and the amplitude coefficient A. The spatial phase component is computed by concatenating the channel-wise average and maximum projections, followed by a 7×7 convolution. This produces the spatial phase map $\theta_{s}$. Accordingly, the method should be interpreted as a classical phase-based trigonometric attention module, rather than as a form of quantum computing or quantum advantage.

The total phase is defined as

(3) $$\theta =\theta_{c}+\theta_{s}$$

To enrich the phase representation, a trigonometric expansion with a second harmonic term is applied:

(4) $$f(\theta )=cos(\theta )+sin(\theta )+0.5\left(cos(2\theta )+sin(2\theta )\right)$$

The resulting modulation term is then applied to the feature map together with the amplitude coefficient:

(5) Q=A⊙X⊙f(θ)

where ⊙ denotes element-wise multiplication. The modulated output is scaled by a learnable LayerScale parameter γ and added back to the input through a residual connection:

(6) Y=X+γQ

**Figure 2:**
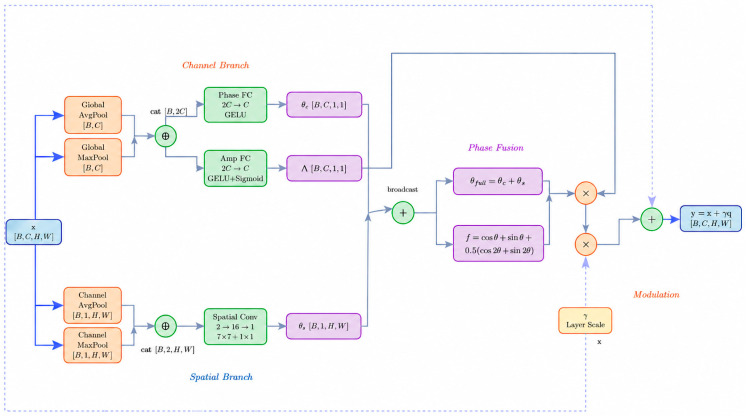
Detailed structure of the Trigonometric Phase Attention (TPA) block. The figure illustrates how channel and spatial phase components are generated, expanded through trigonometric functions, and combined through residual modulation.

Unlike conventional attention modules that mainly rely on direct sigmoid-based reweighting, the proposed design recalibrates feature maps through nonlinear trigonometric modulation, enabling a richer interaction between channel context, spatial context, and residual feature refinement. The detailed structure of the TPA block is shown in Figure 2.

### 3.3. Training Strategy

TPA-ConvNeXt is initialized with a ConvNeXt-Small backbone pre-trained on ImageNet. To adapt these pre-trained representations to medical imaging data in a stable way, we combine layer-wise learning rate decay (LLRD) with a cosine learning rate schedule preceded by a linear warmup phase.

In the LLRD setting, earlier layers are updated with smaller learning rates, while later layers are assigned larger ones. The learning rate for layer l is defined as

(7) $$\eta_{l}=\eta_{0}\cdot \alpha^{d_{l}}$$

where $\eta_{0}$ is the base learning rate, α is the decay factor, and $d_{l}$ represents the depth of the layer. This strategy helps preserve the general visual representations learned by the backbone’s early layers.

During the initial training stage, a linear warmup is applied to avoid abrupt weight updates. After warmup, the learning rate follows a cosine decay schedule:

(8) $$\eta_{t}=\eta_{min}+\frac{1}{2}(\eta_{0}-\eta_{min})\left(1+cos\left(\frac{\pi t}{T}\right)\right)$$

**Figure 3:**
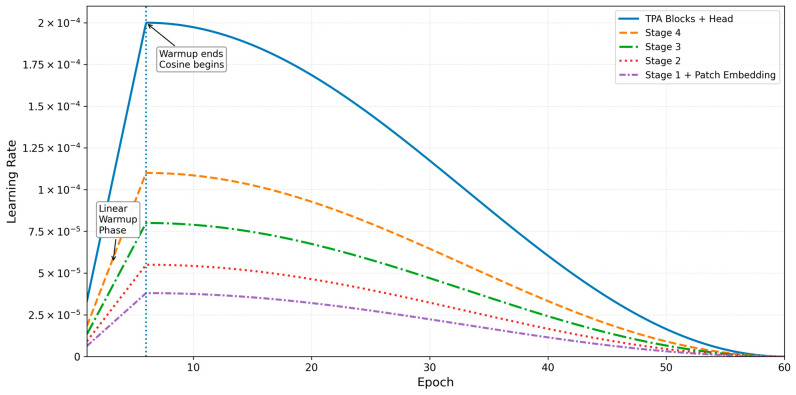
Linear warmup, cosine decay schedule, and layer-wise learning rate distribution used for TPA-ConvNeXt. Earlier layers are updated with smaller learning rates to preserve pre-trained representations.

The linear warmup, cosine decay, and layer-wise learning rate decay work together throughout training. Higher learning rates are used for later layers, while earlier layers are updated more conservatively. This adapts to pre-trained features more stably and reduces the risk of disrupting useful low-level representations. The applied learning rate schedule and the layer-wise distribution are shown in Figure 3.

The final training configuration was selected empirically based on preliminary experiments rather than an exhaustive hyperparameter search. In particular, the decay factor of 0.65 provided a practical balance between preserving early pretrained representations and allowing sufficient adaptation in deeper layers. Similarly, a 5-epoch warmup produced more stable early optimization than shorter warmup settings, whereas longer warmup schedules did not provide a consistent additional benefit. These choices were therefore retained in the final configuration as stable empirical settings for retinal image transfer learning.

## 4. Experimental Setup

This section describes the datasets, preprocessing steps, training configuration, and evaluation metrics used to assess the proposed TPA-ConvNeXt architecture. To examine the generalization ability of the model in a comprehensive way, experiments were carried out on multiple datasets that include both fundus photographs and optical coherence tomography (OCT) images. The proposed architecture was implemented in Python 3.10 using PyTorch 2.2, a deep-learning framework. All experiments were conducted on a workstation equipped with an NVIDIA GeForce RTX 3090 GPU (24 GB VRAM), an AMD Ryzen 9 5950X CPU, and 64 GB RAM. 

### 4.1. Datasets

To evaluate the performance and generalization capability of the proposed method, we used retinal imaging datasets from two main modalities: fundus photography and OCT. These datasets are widely used in the literature and provide valuable benchmarks for AMD detection and retinal disease classification. For the OCT benchmarks, we retained the predefined train/test partitions distributed with the original public dataset releases in order to preserve comparability with prior studies. Therefore, our evaluation followed the official benchmark protocols rather than introducing new custom splits. Because patient identifiers are not uniformly exposed in all released file structures, we did not perform an additional patient-level re-partitioning beyond the provided public splits. Possible subject-level overlap, where relevant, should therefore be interpreted as a limitation of the underlying dataset protocols rather than a source of leakage introduced by our implementation. To further address possible data leakage concerns, we performed an additional split-audit analysis across the datasets used in this study. The audit summarized the evaluation protocol, patient-identifier availability, filename-level overlap, and exact MD5 hash-based duplicate overlap where applicable. For OCT2017, we used the original released Kermany et al. benchmark folder structure without manual modification or custom re-splitting. The official dataset documentation describes the OCT images as being split into training and testing sets from independent patients and labels the images using a disease-randomized patient ID-image number format. Nevertheless, our MD5-based audit identified 45 exact duplicate images between the released OCT2017 training and test folders, corresponding to 4.50% of the 1000-image test set. Therefore, we additionally evaluated the trained model on a cleaned OCT2017 test set after removing these duplicate images.

#### 4.1.1. HYAMD (High-Resolution Fundus AMD Dataset)

HYAMD is a high-resolution fundus image dataset designed for age-related macular degeneration detection [33]. It contains a total of 1560 color fundus images collected from 325 patients and includes both AMD and non-AMD cases. The images were labeled through clinical assessment, and AMD diagnosis was supported by OCT and other ophthalmic imaging methods. HYAMD is the first openly accessible Israeli fundus dataset developed specifically for AMD classification.

In this study, HYAMD was used as the main benchmark for evaluating model performance on fundus images and for obtaining statistically robust results through 5-fold cross-validation.

#### 4.1.2. OCTDL (Optical Coherence Tomography Dataset for Deep Learning)

OCTDL is an open-access OCT dataset containing more than 2000 B-scan images covering multiple retinal pathologies [34]. In addition to AMD, it includes labels for diabetic macular edema (DME), epiretinal membrane (ERM), retinal artery occlusion (RAO), retinal vein occlusion (RVO), and vitreomacular interface disease (VID). The images were acquired through raster scanning centered on the fovea and were interpreted by expert retinal clinicians.

This dataset was used to evaluate the model on a multi-class OCT classification task.

#### 4.1.3. OCT2017 (Kermany et al. Benchmark)

OCT2017 is one of the most widely used OCT benchmarks in deep learning studies on retinal disease classification [35]. The dataset contains more than 84,000 OCT B-scan images and is organized into four major categories: normal retina, diabetic macular edema (DME), drusen, and choroidal neovascularization (CNV).

Because of its large scale and broad use in the literature, this dataset was included to assess the general OCT classification performance of the proposed model in a more extensive setting. In this study, OCT2017 was used exactly according to its originally released benchmark structure. No custom train/test re-splitting or manual file rearrangement was performed. Although the original dataset documentation reports independent-patient train/test partitions, our additional MD5-based audit detected a small number of exact duplicate images in the local released train/test structure. For this reason, OCT2017 was interpreted as a benchmark-level evaluation dataset, and an additional clean-test analysis was conducted after removing the detected duplicate test images.

#### 4.1.4. MAK1_OCT

The MAK1_OCT dataset used in this study is based on a private OCT dataset introduced by Yusufoğlu et al. in 2024 [27]. It was collected at the Ophthalmology Department of Elazığ Fethi Sekin City Hospital in Türkiye. The dataset contains 2316 OCT images obtained from 653 eyes belonging to 256 different participants. It includes three classes. The Dry AMD class contains 653 images from 89 patients, the Wet AMD class includes 743 images from 82 patients, and the Normal class consists of 920 images from 85 healthy individuals.

The images were acquired in a clinical setting using high-resolution OCT devices. Dry AMD cases show retinal thinning and drusen accumulation, whereas wet AMD cases include pathological findings such as choroidal neovascularization and fluid accumulation. Since the dataset covers both exudative and non-exudative forms of AMD, it provides a clinically meaningful and well-structured benchmark. In this work, MAK1_OCT was used for a detailed evaluation of the classification performance of the proposed model.

#### 4.1.5. AMDNet23 Fundus Dataset

To further assess external generalization in fundus imaging, we additionally used the AMDNet23 fundus dataset [18]. This dataset is based on a four-class AMD grading setting compiled from public fundus image sources and provides a useful benchmark for evaluating cross-dataset robustness beyond HYAMD. In this study, AMDNet23 was used in three complementary ways: (i) as an external validation set for a model trained on HYAMD, (ii) as an independent four-class fundus benchmark using its own released train/validation split, and (iii) as part of a pooled binary experiment in combination with HYAMD. These additional experiments were designed to evaluate whether the proposed framework can generalize beyond a single fundus dataset and remain stable under broader data heterogeneity.

### 4.2. Common Dataset Characteristics

Across all datasets, the images are centered on the retina and reflect retinal structural information. The OCT datasets consist of B-scan cross-sectional retinal images, while the fundus datasets contain color photographs of the retinal surface. In all cases, labels were assigned based on clinical evaluation.

### 4.3. Clinical Relevance of the Datasets

Fundus and OCT images provide complementary views of retinal pathology. Fundus images highlight the surface appearance of the retina and visible lesions in the macular region, whereas OCT B-scans reveal the cross-sectional microstructure of retinal layers. Evaluating the same model on both modalities makes it possible to assess not only classification performance, but also modality robustness.

### 4.4. Preprocessing and Data Augmentation

A standardized preprocessing and augmentation pipeline was applied before training in order to account for structural characteristics of retinal images and variations in image acquisition conditions. The goal was to create a consistent input distribution across both fundus and OCT datasets while improving the generalization ability of the model.

For fundus images, the first step was contrast enhancement using Contrast-Limited Adaptive Histogram Equalization (CLAHE). This operation helps make low-contrast lesions in the macular region more visible and improves the representation of vessel structures and drusen-like formations. In this study, the CLAHE clip limit was set to 2.5. Since OCT images tend to have a more homogeneous intensity distribution, CLAHE was applied only to fundus images.

After contrast enhancement, all images were resized to 224 × 224 pixels. This resolution matches the input requirements of the ConvNeXt-based backbone. During training, random cropping was used to improve robustness to small positional shifts.

To reduce overfitting and improve generalization, random data augmentation was applied during training. This included random rotation within ±15°, horizontal flipping when anatomically appropriate, and color-based perturbations such as brightness and contrast jitter. For OCT images, where anatomical symmetry is more limited, flipping operations were applied in a controlled manner.

Finally, all images were normalized using the ImageNet mean and standard deviation values. This step helps align the input distribution with the pretrained backbone and supports more stable transfer learning.

**Figure 4:**
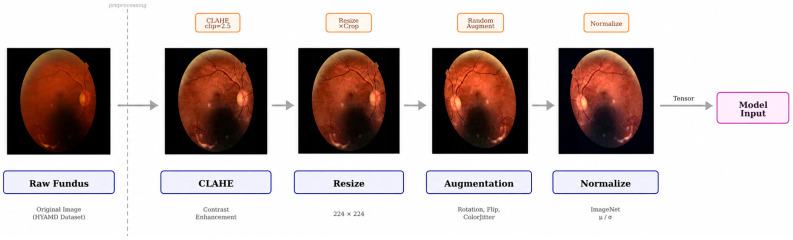
Preprocessing pipeline used in TPA-ConvNeXt. The figure shows contrast enhancement with CLAHE, resizing, random data augmentation, and ImageNet normalization for fundus images.

The overall preprocessing and augmentation pipeline is illustrated in Figure 4.

### 4.5. Training Details

TPA-ConvNeXt was initialized with a ConvNeXt-Small backbone pretrained on ImageNet. All experiments were implemented using the PyTorch 2.2 deep learning framework. AdamW was used as the optimizer throughout training, and the weight decay value was set to $1\times 10^{-4}$. The base learning rate was fixed at $2\times 10^{-4}$, and this value was assigned to the newly added TPA blocks and the classification head.

To adapt pretrained layers in a stable way, layer-wise learning rate decay (LLRD) was applied. The decay factor was set to λ=0.65, which allowed earlier layers to be updated more conservatively than later ones. A linear warmup was used during the first 5 epochs, followed by a cosine decay schedule. The maximum number of training epochs was set to 60.

The mini-batch size was 32. Early stopping was also used during training. If no improvement was observed in the validation Macro F1 score, training was stopped. This helped reduce overfitting and supported better generalization.

Cross-entropy loss was used as the objective function. To improve training stability, label smoothing was applied with a smoothing factor of 0.05. Based on the ablation analysis, class weighting did not improve performance and was therefore removed from the final configuration.

All experiments were conducted on an NVIDIA RTX 4080 SUPER GPU. Although training time and runtime varied depending on the dataset, the 5-fold cross-validation process was performed independently for each fold. A fixed random seed was used to reduce the effect of randomness.

For the benchmark comparisons in Table 1, all baseline models were trained under the same data split, preprocessing pipeline, augmentation settings, evaluation metrics, and stopping criterion to ensure a fair comparison. Architecture-specific modifications were limited to replacing the final classifier head and adapting optimization grouping when required by the backbone structure.

### 4.6. Evaluation Metrics

The performance of TPA-ConvNeXt was assessed using several standard classification metrics. Since retinal image datasets often suffer from class imbalance, accuracy alone was not considered sufficient. For this reason, Macro F1 was selected as the primary evaluation metric. For the single-split OCT experiments, 95% confidence intervals were additionally reported for Accuracy, Macro F1, Weighted F1, and ROC-AUC. The confidence intervals were estimated using bootstrap resampling of the validation-set predictions.

#### 4.6.1. Accuracy

Accuracy measures the ratio of correctly classified samples to the total number of samples:

(9) $$Accuracy=\frac{TP+TN}{TP+TN+FP+FN}$$

where TP, TN, FP, and FN denote true positives, true negatives, false positives, and false negatives, respectively. Although accuracy provides an overall performance estimate, it can be misleading when class distributions are imbalanced.

#### 4.6.2. Precision, Recall, and F1-Score

For each class, precision and recall were computed as follows:

(10) $$Precision=\frac{TP}{TP+FP}$$

(11) $$Recall=\frac{TP}{TP+FN}$$

The F1-score is defined as the harmonic mean of precision and recall:

(12) $$F1=2\cdot \frac{Precision\cdot Recall}{Precision+Recall}$$

#### 4.6.3. Macro F1 and Weighted F1

In multi-class settings, Macro F1 is preferred when all classes are considered equally important. It is calculated as the arithmetic mean of class-wise F1-scores:

(13) $$\mathrm{Macro}\;F1=\frac{1}{C}\sum_{i=1}^{C}F1_{i}$$

where C is the number of classes. This metric is especially informative in medical datasets because it gives equal weight to minority classes.

Weighted F1, on the other hand, computes the average F1-score by weighting each class according to its proportion in the dataset:

(14) $$\mathrm{Weighted}\;F1=\sum_{i=1}^{C}w_{i}F1_{i}$$

where $w_{i}$ represents the relative frequency of class i.

In this study, Macro F1 was reported as the main performance metric in order to reduce the influence of class imbalance.

#### 4.6.4. ROC-AUC

To measure the discriminative ability of the model in both binary and multi-class settings, the area under the receiver operating characteristic curve (ROC-AUC) was calculated. For multi-class experiments, the one-vs-rest (OvR) approach was used, and the macro average AUC was reported.

## 5. Results

This section presents the performance of the proposed TPA-ConvNeXt model in detail. We first report the single-fold comparison against standard deep learning architectures, then provide the 5-fold cross-validation results, followed by the ablation analysis and multi-dataset generalization results.

### 5.1. Single-Fold Comparison with Standard Architectures

TPA-ConvNeXt was compared with strong CNN- and Transformer-based architectures on the HYAMD dataset. All models were evaluated on Fold 4 under the same training and preprocessing protocol.

The comparison results are presented in Table 1.

In this exploratory single-fold comparison, TPA-ConvNeXt achieved the highest Accuracy and Macro F1 among the evaluated architectures on Fold 4. However, this result should be interpreted as a single-fold benchmark rather than as primary statistical evidence of superiority. Therefore, the main statistical comparison was conducted separately against the closest architectural baseline, ConvNeXt-Small, across the five HYAMD folds.

### 5.2. Five-Fold Cross-Validation Results on HYAMD

To reduce the possibility that the single-fold result was influenced by a favorable split, TPA-ConvNeXt was further evaluated on the HYAMD dataset using 5-fold cross-validation. The results are reported in Table 2.

The mean Macro F1 score was 0.8940 ± 0.0403. The strong result obtained on Fold 4 was not reproduced at the same level across all folds, indicating that performance varied depending on the data split. Even so, the model maintained stable ROC-AUC values with relatively low variance.

Overall, these results indicate that the proposed architecture performs consistently across different train–validation splits, while also highlighting the influence of dataset partitioning on fundus-based AMD classification.

#### Statistical Comparison with ConvNeXt-Small Across Five Folds

**Figure 5:**
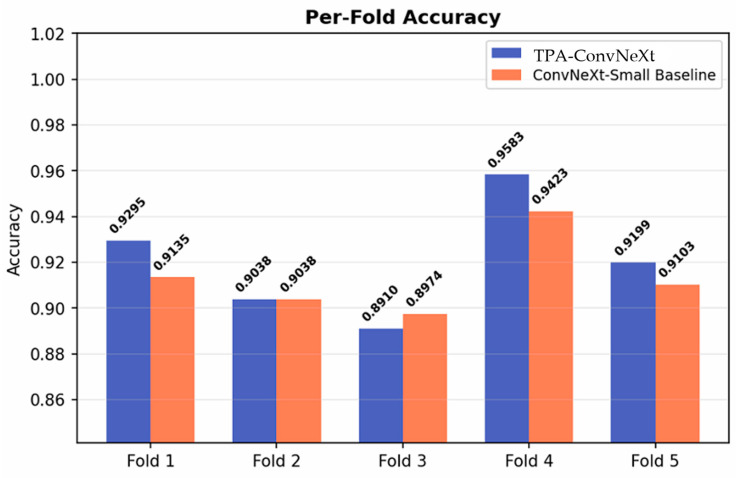
Per-fold accuracy comparison between TPA-ConvNeXt and ConvNeXt-Small on the HYAMD dataset. The difference was numerical and did not reach statistical significance across the five folds.

To complement the fold-wise averages in Table 2, TPA-ConvNeXt was compared with the closest architectural baseline, ConvNeXt-Small, across the same five HYAMD folds. TPA-ConvNeXt achieved a mean accuracy of 0.9205 ± 0.0231, whereas ConvNeXt-Small achieved 0.9135 ± 0.0154. The mean paired accuracy difference was +0.0071, with a 95% confidence interval of [−0.0053, +0.0194]. Since this interval included zero, the observed difference should be interpreted as a small numerical trend rather than a statistically reliable improvement. Figure 5 shows per-fold accuracy scores for TPA-ConvNeXt and ConvNeXt-Small Baseline architectures.

The paired *t*-test did not show statistical significance (t = 1.5795, *p* = 0.1894), and the Wilcoxon signed-rank test also remained non-significant (W = 1.0000, *p* = 0.1408). Therefore, the five-fold comparison does not support a claim of statistically significant superiority over ConvNeXt-Small. Instead, these results indicate that TPA-ConvNeXt provides competitive performance relative to the ConvNeXt-Small backbone, while adding a phase-based trigonometric modulation mechanism that may support feature recalibration in retinal image classification.

### 5.3. Ablation Study

An ablation study was conducted to examine the contribution of each major component in the proposed framework. The results are given in Table 3.

Among all components, warmup had the strongest impact on performance. When warmup was removed, Macro F1 dropped by about 2.8 points. This suggests that pretrained backbones are sensitive to abrupt learning rate changes and that the warmup phase plays a key role in stabilizing fine-tuning.

Removing the Trigonometric attention block led to a smaller but still consistent drop in performance. This indicates that the proposed phase-based modulation contributes positively to the model, even if its effect is subtler than the optimization-related components. The results also show that LLRD, CLAHE, and label smoothing all provide meaningful benefits.

#### Hyperparameter Sensitivity Analysis

To further examine the empirical choice of the LLRD decay factor and warmup length, a limited hyperparameter sensitivity analysis was conducted on the HYAMD Fold 4 validation setting. Two small sensitivity sweeps were performed. In the first sweep, the warmup length was fixed at 5 epochs while the LLRD decay factor was varied. In the second sweep, the LLRD decay factor was fixed at 0.65 while the warmup length was varied.

As shown in Table 4, the decay factor of 0.65 achieved the best performance among the tested decay settings, with a Macro F1 of 0.9555 and an accuracy of 0.9551. A lower decay factor of 0.50 reduced Macro F1 to 0.9385, suggesting that overly conservative updates in earlier layers may limit adaptation. A higher decay factor of 0.80 also performed well but remained slightly lower than 0.65.

The warmup sensitivity analysis also supported the use of a 5-epoch warmup. Removing warmup reduced Macro F1 to 0.9051, confirming that abrupt early learning-rate changes can destabilize fine-tuning. A 3-epoch warmup improved performance compared with no warmup, whereas an 8-epoch warmup did not provide additional benefit. Overall, these results support the selected configuration of an LLRD decay factor of 0.65 and a 5-epoch warmup as a stable empirical setting for TPA-ConvNeXt.

### 5.4. Generalization Across Multiple Datasets

To show that TPA-ConvNeXt is not limited to a single dataset, additional experiments were carried out on multiple OCT datasets. These experiments were designed to assess how well the model generalizes across different class distributions and different clinical acquisition settings.

#### 5.4.1. Results on MAK1_OCT

The results obtained on the private OCT dataset, MAK1_OCT, are presented in Table 5.

The model achieved very strong performance on this three-class AMD classification task. In particular, the ROC-AUC value of 0.9960 indicates an excellent level of class separability.

Class-wise analysis further supports this result. The F1-score was 0.9660 for Dry AMD, 0.9945 for Normal, and 0.9764 for Wet AMD. The near-perfect performance on the Normal class, together with the strong results on the pathological classes, suggests that the model can capture clinically relevant distinctions.

#### 5.4.2. Multi-Class Results on OCTDL

TPA-ConvNeXt was also evaluated on the OCTDL dataset, which involves a seven-class retinal pathology classification task. Results on the OCTDL dataset are given in Table 6.

This dataset represents a more challenging classification problem because it includes several different retinal diseases. On OCTDL, the model achieved an accuracy of 0.9564 [95% CI: 0.9370–0.9734], a Macro F1 of 0.9387 [95% CI: 0.9011–0.9668], and an ROC-AUC of 0.9722 [95% CI: 0.9496–0.9907]. Considering the limited number of samples in some classes, particularly RAO and RVO, these results remain encouraging. They suggest that the proposed phase-based mechanism can still preserve useful representations even in small or imbalanced categories.

#### 5.4.3. Binary Classification on OCTDL_AMD

The model was further tested on the AMD vs. Non-AMD subset of the OCTDL dataset. Binary classification results on OCTDL_AMD are given in Table 7.

In this binary setting, the model achieved near-perfect discrimination. On the binary OCTDL_AMD setting, TPA-ConvNeXt achieved an accuracy of 0.9855 [95% CI: 0.9734–0.9952], a Macro F1 of 0.9849 [95% CI: 0.9722–0.9950], and an ROC-AUC of 0.9927 [95% CI: 0.9798–0.9997].

#### 5.4.4. OCT2017 Clean-Test Evaluation After MD5 Duplicate Removal

To evaluate whether exact duplicate images between the released OCT2017 training and test folders affected the reported performance, an additional clean-test analysis was conducted. First, all OCT2017 training images were hashed using MD5. Each test image was then compared with the training hash list. This analysis identified 45 exact duplicate images in the test set, corresponding to 4.50% of the original 1000 test images. Most duplicate images belonged to the DME class, with 41 duplicates, while 4 duplicates were found in the Normal class. No duplicates were detected in the CNV or Drusen classes.

After removing these duplicate images, the cleaned OCT2017 test set contained 955 images. The same trained checkpoint was evaluated on both the original and cleaned test sets without any additional training. The results remained practically unchanged. Accuracy was 0.9990 on both the original and cleaned test sets, while Macro F1 changed only from 0.9990 to 0.9989. These findings indicate that the detected duplicate samples had a negligible effect on the OCT2017 performance. The results of OCT2017 original and clean-test evaluation after MD5 duplicate removal are given in Table 8.

#### 5.4.5. External Fundus Validation and Cross-Dataset Experiments

To further assess whether the proposed framework generalizes beyond a single internal fundus benchmark, we conducted three additional fundus experiments using AMDNet23. First, we performed an external validation experiment in which the model was trained on HYAMD and tested on the AMDNet23 validation set after converting the labels to a binary AMD versus non-AMD setting. Second, we trained and evaluated the model directly on AMDNet23 under its native four-class setting. Third, we conducted a pooled binary experiment on the combined HYAMD + AMDNet23 dataset to examine performance under broader fundus-domain heterogeneity. The results are summarized in Table 9.

In the external validation setting (E1), the model achieved 0.9350 accuracy, 0.9312 Macro F1, and 0.9803 ROC-AUC, indicating that the learned representation transfers well from HYAMD to an independent fundus dataset. In the native four-class AMDNet23 experiment (E2), the model achieved 0.9825 accuracy and 0.9825 Macro F1, showing that the proposed framework also performs strongly in a more fine-grained fundus classification setting. Finally, in the pooled binary experiment on the merged HYAMD + AMDNet23 data (E3), the model achieved 0.9570 accuracy and 0.9466 Macro F1. Taken together, these experiments strengthen the claim that TPA-ConvNeXt is not restricted to a single fundus source and can remain robust across different fundus data distributions.

#### 5.4.6. Explainability Analysis with Grad-CAM

**Figure 6:**
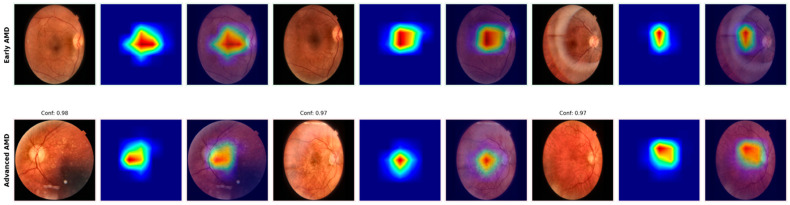
Representative Grad-CAM visualizations for fundus images from the Early AMD and Advanced AMD classes. The maps show that TPA-ConvNeXt primarily focuses on the central retinal and macular region during classification.

To provide a qualitative explanation of the model’s decision process, we generated Grad-CAM visualizations for representative fundus images from the Early AMD and Advanced AMD classes. As shown in Figure 6, the model consistently concentrated its attention on the central retinal and macular region rather than on peripheral background areas. In Early AMD cases, the highlighted regions were generally more localized, whereas in Advanced AMD cases the activated areas tended to be broader and more intense. Although Grad-CAM does not provide lesion-level segmentation, these visualizations support that the proposed model bases its predictions on clinically plausible retinal regions and therefore improve the interpretability of the reported classification results.

**Figure 7:**
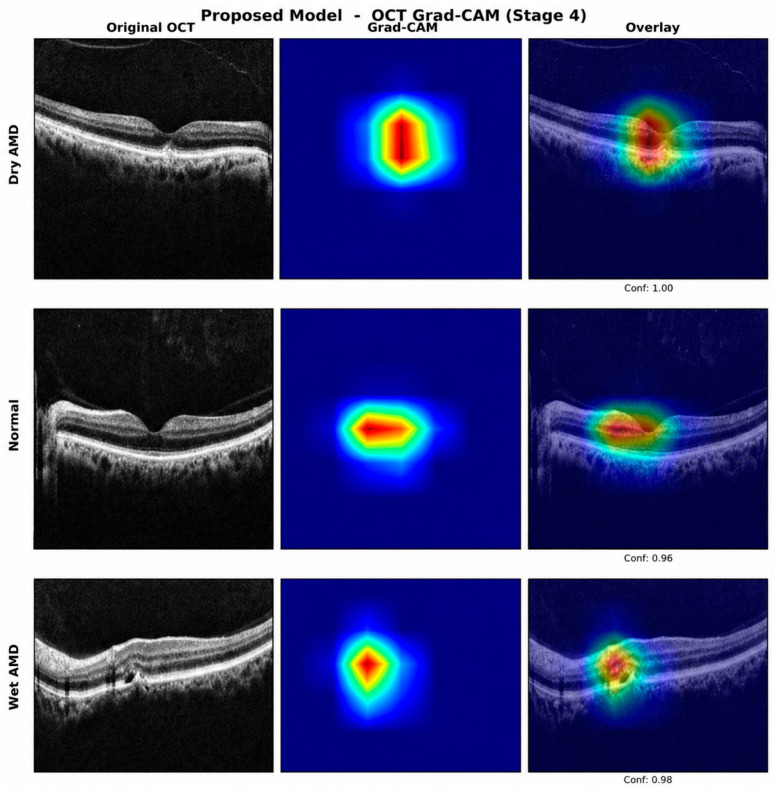
OCT Grad-CAM visualization of the proposed model using representative MAK1_OCT samples. Each row shows the original OCT image, the Grad-CAM heatmap, and the overlay map for one class. The Grad-CAM maps were generated from the Stage 4 output feature maps. The highlighted regions indicate that the model mainly focuses on the foveal region and retinal layer abnormalities, which are clinically relevant for OCT-based AMD classification.

In response to the concern that explainability analysis should also cover OCT images, we additionally generated Grad-CAM visualizations for representative OCT samples from the MAK1_OCT dataset. The target layer was the output of the fourth ConvNeXt stage, corresponding to the final 7 × 7 feature map before global pooling. As shown in Figure 7, the activation maps were concentrated around clinically meaningful retinal structures, including the foveal region and abnormal retinal layer patterns. For dry AMD and wet AMD examples, the highlighted regions overlapped with areas showing structural irregularities in the retinal layers. In the normal OCT example, the model mainly attended to the central retinal contour and foveal depression. These findings suggest that the model does not rely only on peripheral or background artifacts, but focuses on diagnostically relevant retinal regions in OCT images as well.

### 5.5. Computational Complexity and Runtime Analysis

To evaluate the computational overhead introduced by the proposed phase-based attention blocks, we compared TPA-ConvNeXt with the ConvNeXt-Small baseline in terms of parameter count, MACs, FLOPs, and inference latency. All runtime measurements were performed on an NVIDIA RTX 4080 SUPER GPU using 224 × 224 input images.

As shown in Table 10, TPA-ConvNeXt contained 51.82 million parameters, whereas the ConvNeXt-Small baseline contained 49.46 million parameters. The proposed attention blocks added 2.36 million parameters, corresponding to a modest parameter increase over the baseline. In terms of computational operations, TPA-ConvNeXt required 8.71 G MACs and approximately 17.41 G FLOPs, while ConvNeXt-Small required 8.70 G MACs and approximately 17.39 G FLOPs. This indicates that the proposed attention mechanism introduced only a very small FLOP overhead.

The inference latency was also measured. For single-image inference, TPA-ConvNeXt required 13.76 ± 6.46 ms, compared with 10.89 ± 5.30 ms for ConvNeXt-Small. In batch-32 inference, which provides a more stable estimate of throughput, TPA-ConvNeXt required 1.34 ± 0.39 ms per image, whereas ConvNeXt-Small required 1.09 ± 0.03 ms per image. These results show that the proposed attention mechanism adds a moderate parameter and latency overhead, but the overall model remains computationally practical for GPU-based retinal image classification.

### 5.6. Overall Assessment

Taken together, the results show that TPA-ConvNeXt achieves strong discrimination on fundus images, balanced performance on multi-class OCT problems, and near-perfect results in binary AMD detection. Beyond the original HYAMD and OCT experiments, the additional AMDNet23 results further show that the proposed framework generalizes well across independent fundus datasets and remains robust under pooled cross-dataset settings. The newly added statistical comparison showed a small numerical accuracy difference relative to ConvNeXt-Small across the five HYAMD folds; however, this difference did not reach statistical significance under paired testing. Finally, the Grad-CAM and runtime analyses show that the proposed framework is not only accurate, but also interpretable and computationally practical.

## 6. Discussion

The proposed TPA-ConvNeXt architecture showed competitive performance across both fundus and OCT datasets. Overall, the results suggest that phase-based feature modulation may support discriminative representation learning in retinal image classification.

In the exploratory Fold 4 comparison, TPA-ConvNeXt achieved higher Accuracy and Macro F1 than the evaluated CNN and Transformer baselines. However, because this comparison was limited to a single fold, it should be interpreted as supportive evidence rather than definitive proof of superiority.

The ablation study revealed that warmup was the most critical component in the final system. When warmup was removed, Macro F1 dropped by nearly 2.8 points. This indicates that pretrained backbones may be easily disrupted when adapted to medical imaging data with large updates at the beginning of training. A more gradual transition appears to be essential for stable fine-tuning. A similar trend was observed for LLRD, which helped protect early-layer representations and improved optimization stability. The additional hyperparameter sensitivity analysis further supports this interpretation. The selected decay factor of 0.65 achieved the best result among the tested LLRD settings, and the 5-epoch warmup clearly outperformed no warmup and shorter or longer warmup alternatives in the tested one-fold setting.

The TPA block provided a measurable gain in the Fold 4 ablation study, although its effect was smaller than the contribution of warmup. This suggests that the architectural component may complement the optimization strategy, but the current experiments do not fully isolate all interaction effects between components.

The multi-dataset results also provide important evidence of modality robustness. When the results from fundus and OCT datasets are considered together, TPA-ConvNeXt appears to generalize well across different retinal imaging techniques. The strong performance on a large benchmark such as OCT2017 further suggests that the model is not limited to small-scale or institution-specific datasets.

An important extension of the present study is the additional external fundus validation using AMDNet23. While HYAMD remained the main benchmark for internal 5-fold analysis, the external experiment showed that a model trained on HYAMD could still achieve 0.9312 Macro F1 and 0.9803 ROC-AUC on AMDNet23. This result strengthens the interpretation that the learned representation is not restricted to a single fundus dataset. The direct four-class experiment on AMDNet23 and the pooled binary experiment on the combined HYAMD + AMDNet23 data further support the cross-dataset robustness of the proposed framework. The addition of OCT Grad-CAM examples further supports the cross-modality interpretability of the model. While fundus Grad-CAM maps emphasized macular regions and visible lesion patterns, OCT Grad-CAM maps highlighted the foveal contour and retinal layer abnormalities. This suggests that the model adapts its attention behavior to modality-specific anatomical structures.

The statistical comparison with ConvNeXt-Small should be interpreted cautiously. Although TPA-ConvNeXt showed a small numerical increase in mean accuracy across the five HYAMD folds, this difference was not statistically significant, and the confidence interval included zero. Therefore, the proposed model should not be interpreted as a statistically proven superior replacement for ConvNeXt-Small on HYAMD alone. Rather, its contribution lies in introducing a phase-based trigonometric attention mechanism, combining it with a stable fine-tuning strategy, and evaluating the resulting framework across fundus and OCT datasets with external validation, ablation, explainability, and computational cost analyses.

The computational analysis also shows that the proposed attention block increases the parameter count from 49.46 M to 51.82 M, while adding only a negligible FLOP overhead. The main runtime cost is reflected in inference latency, especially in single-image evaluation, which is expected because the attention block includes trigonometric operations. However, batch inference remained efficient at 1.34 ms per image on an RTX 4080 SUPER GPU. Therefore, the proposed module provides additional feature modulation capacity with a limited computational burden.

At the same time, several limitations should be acknowledged. First, the 5-fold cross-validation results on HYAMD showed noticeable variation between folds, indicating that performance remains influenced by data partitioning and class composition. Second, for the OCT benchmarks we retained the predefined train/test folders distributed with the original public releases in order to preserve comparability with prior studies. Because patient identifiers are not uniformly available in all public file structures, additional patient-level re-splitting was not performed, and possible subject-level overlap should be interpreted as a limitation of the underlying dataset protocols rather than of our implementation itself. Third, although the TPA block provided a consistent benefit, the ablation results showed that optimization-level components such as warmup contributed a larger gain than the architectural block alone. Fourth, the present study was conducted on 2D images only, and broader prospective, multi-center validation will still be necessary before clinical deployment can be considered. Finally, although the runtime results were practical on modern GPU hardware, real-world translation will also require validation under device heterogeneity, workflow integration constraints, and explainability requirements in clinical settings. A further limitation concerns the reliability of public benchmark splits. OCT2017 was used according to the original released Kermany et al. benchmark structure, whose documentation describes independent-patient train/test partitions. However, our additional MD5-based audit detected 45 exact duplicate images between the released training and test folders. To assess the impact of this issue, we repeated the evaluation after removing these duplicate test images. The clean-test performance was nearly identical to the original result, suggesting that the duplicate samples had a negligible effect on the reported OCT2017 performance. Nevertheless, OCT2017 results should still be interpreted as benchmark-level evidence rather than as newly constructed patient-level external validation. Future work should prioritize multi-center datasets with explicit patient identifiers, duplicate-free public releases, and strictly patient-level external test cohorts.

## 7. Conclusions

In this study, we proposed TPA-ConvNeXt, a retinal image classification framework built on a ConvNeXt-Small backbone and enhanced with a phase-based trigonometric attention mechanism. The proposed TPA block recalibrates feature maps through learnable phase and amplitude modulation, while the accompanying fine-tuning strategy based on layer-wise learning rate decay, linear warmup, and cosine scheduling improves optimization stability on medical image data.

The experimental results showed that the proposed framework achieved strong performance on both fundus and OCT datasets. On HYAMD, 5-fold cross-validation demonstrated overall competitive performance, while the additional statistical analysis showed that the difference from the ConvNeXt-Small baseline was not statistically significant. External fundus validation on AMDNet23 further strengthened the cross-dataset evaluation, and the results obtained on MAK1_OCT, OCTDL, and OCT2017 confirmed that the framework generalizes well across multiple OCT settings.

The ablation study showed that warmup had the largest optimization effect, whereas the proposed TPA block contributed a smaller but consistent representational gain. In addition, Grad-CAM visualizations indicated that the model focused on clinically relevant retinal regions, and the complexity analysis showed that the framework remained computationally practical.

Overall, TPA-ConvNeXt provides a competitive, interpretable, and computationally practical framework for retinal disease classification across fundus and OCT modalities. However, its improvement over ConvNeXt-Small should be interpreted cautiously, and broader patient-level multi-center validation remains necessary.

## Figures and Tables

**Table 1:** Exploratory single-fold comparison of different architectures on Fold 4 of the HYAMD dataset.

Model	Accuracy	Macro F1	Weighted F1	ROC-AUC
ConvNeXt-Small	0.9359	0.9389	0.9358	0.9728
ConvNeXt-Tiny	0.9231	0.9258	0.9218	0.9701
EfficientNet-B0	0.9167	0.8990	0.9182	0.9805
ResNet101	0.8910	0.8915	0.8917	0.9510
DenseNet121	0.8878	0.8848	0.8895	0.9613
MobileNetV3-Large	0.8942	0.8846	0.8935	0.9577
VGG19	0.8654	0.8771	0.8684	0.9568
ResNet50	0.8814	0.8762	0.8838	0.9677
ViT-B/16	0.8462	0.8305	0.8489	0.9425
TPA-ConvNeXt	0.9583	0.9636	0.9581	0.9605

**Table 2:** Five-fold cross-validation results of TPA-ConvNeXt on HYAMD.

Fold	Accuracy	Macro F1	ROC-AUC
Fold 1	0.9295	0.9051	0.9443
Fold 2	0.9038	0.8428	0.9419
Fold 3	0.8910	0.8719	0.9359
Fold 4	0.9583	0.9636	0.9677
Fold 5	0.9199	0.8864	0.9348
Mean	0.9205	0.8940	0.9449
Std	0.0231	0.0403	0.0119

**Table 3:** Ablation analysis of TPA-ConvNeXt on Fold 4.

Configuration	Macro F1	ΔF1
Full Model	0.9645	+0.0060
w/o TrigonometricAttn	0.9553	−0.0032
w/o LLRD	0.9523	−0.0062
w/o CLAHE	0.9501	−0.0085
w/o Label Smoothing	0.9463	−0.0122
w/o Warmup	0.9304	−0.0281

**Table 4:** Hyperparameter sensitivity analysis for LLRD decay factor and warmup length on HYAMD.

Sensitivity Setting	Configuration	Macro F1	Accuracy
LLRD decay sweep	decay = 0.50, warmup = 5	0.9385	0.9359
LLRD decay sweep	decay = 0.65, warmup = 5	0.9555	0.9551
LLRD decay sweep	decay = 0.80, warmup = 5	0.9529	0.9519
Warmup sweep	decay = 0.65, warmup = 0	0.9051	0.9231
Warmup sweep	decay = 0.65, warmup = 3	0.9322	0.9295
Warmup sweep	decay = 0.65, warmup = 5	0.9507	0.9423
Warmup sweep	decay = 0.65, warmup = 8	0.9365	0.9391

**Table 5:** Performance results of TPA-ConvNeXt on the MAK1_OCT dataset with 95% confidence intervals.

Metric	Value [95% CI]
Accuracy	0.9806 [0.9677, 0.9914]
Macro F1	0.9790 [0.9644, 0.9911]
Weighted F1	0.9807 [0.9678, 0.9914]
ROC-AUC	0.9960 [0.9899, 0.9995]

Note: Confidence intervals were calculated at the 95% level using the validation predictions. The validation set contained 464 images.

**Table 6:** Performance results of TPA-ConvNeXt on the OCTDL dataset with 95% confidence intervals.

Metric	Value [95% CI]
Accuracy	0.9564 [0.9370, 0.9734]
Macro F1	0.9387 [0.9011, 0.9668]
Weighted F1	0.9562 [0.9359, 0.9734]
ROC-AUC	0.9722 [0.9496, 0.9907]

Note: Confidence intervals were calculated at the 95% level using the validation predictions. The validation set contained 413 images.

**Table 7:** Binary classification results on OCTDL_AMD with 95% confidence intervals.

Metric	Value [95% CI]
Accuracy	0.9855 [0.9734, 0.9952]
Macro F1	0.9849 [0.9722, 0.9950]
Weighted F1	0.9855 [0.9734, 0.9952]
ROC-AUC	0.9927 [0.9798, 0.9997]

Note: Confidence intervals were calculated at the 95% level using the validation predictions. The validation set contained 413 images.

**Table 8:** OCT2017 original and clean-test evaluation after MD5 duplicate removal.

Test Set	N Images	Removed Duplicates	Accuracy	Macro F1	Weighted F1	ROC-AUC
Original OCT2017 test	1000	0	0.9990	0.9990	0.9990	0.9999
Clean OCT2017 test	955	45	0.9990	0.9989	0.9990	0.9999
Delta	−45	45	−0.0000	−0.0001	−0.0000	−0.0000

**Table 9:** Additional fundus experiments using AMDNet23 for external validation, independent four-class evaluation, and pooled binary evaluation.

Experiment	Training Set	Test Set	Classes	Accuracy	Macro/Binary F1	Weighted F1	ROC-AUC	Precision	Recall	Time (min)
E1	HYAMD (binary)	AMDNet23 valid (binary)	2	0.9350	0.9312	0.9348	0.9803	0.9888	0.8800	17.33
E2	AMDNet23 train (4-class)	AMDNet23 valid (4-class)	4	0.9825	0.9825	0.9825	0.9956	0.9836	0.9825	27.04
E3	HYAMD + AMDNet23 (binary)	HYAMD + AMDNet23 (binary)	2	0.9570	0.9466	0.9570	0.9696	0.9466	0.9466	77.81

**Table 10:** Computational complexity and inference time comparison between TPA-ConvNeXt and ConvNeXt-Small.

Model	Params (M)	TPA Params (M)	MACs (G)	FLOPs (G)	Single Image (ms)	Batch-32 (ms/img)
ConvNeXt-Small	49.46	0.00	8.70	17.39	10.89 ± 5.30	1.09 ± 0.03
TPA-ConvNeXt	51.82	2.36	8.71	17.41	13.76 ± 6.46	1.34 ± 0.39
Overhead	+2.36	+2.36	+0.01	+0.02	+2.87	+0.25

Note: FLOPs were estimated as approximately 2 × MACs. Runtime was measured on an NVIDIA RTX 4080 SUPER GPU using 224 × 224 input images.

## Data Availability

The datasets used in this study are publicly available and can be accessed through the following sources: HYAMD dataset: https://physionet.org/content/hillel-yaffe-fundus-amd/1.0.0/; OCTDL dataset: https://www.kaggle.com/datasets/orvile/octdl-optical-coherence-tomography-dataset; OCT2017 dataset: https://data.mendeley.com/datasets/rscbjbr9sj/1; AMDNet23 dataset: https://data.mendeley.com/datasets/yj35kjgrv3/1; (accessed on: 7 January 2026) All datasets employed in this work are publicly accessible and were obtained from the above repositories.

## Abbreviations

The following abbreviations are used in this manuscript:

TPA	Trigonometric Phase Attention
LLRD	Layer-wise Learning Rate Decay
OCT	Optical Coherence Tomography
AMD	Age-related Macular Degeneration
CNNs	Convolutional Neural Networks
ViT	Vision Transformer
